# A prospective study to evaluate the intra-individual reproducibility of bone scans for quantitative assessment in patients with metastatic prostate cancer

**DOI:** 10.1186/s12880-018-0257-5

**Published:** 2018-05-04

**Authors:** Mariana Reza, Reza Kaboteh, May Sadik, Anders Bjartell, Per Wollmer, Elin Trägårdh

**Affiliations:** 1 Department of Clinical Physiology and Nuclear Medicine, Skåne University Hospital, Inga Marie Nilssons gata 49, SE-205 02 Malmö, Sweden; 2 Department of Translational Medicine, Lund University, Malmö, Sweden; 3 Department of Molecular and Clinical Medicine, Sahlgrenska University Hospital, Gothenburg, Sweden; 4 Department of Urology, Skåne University Hospital, Malmö, Sweden

**Keywords:** Reproducibility, Prostate cancer, Bone metastasis, Bone scan quantitative analysis, Bone scan index

## Abstract

**Background:**

The Bone Scan Index (BSI) is used to quantitatively assess the total tumour burden in bone scans of patients with metastatic prostate cancer. The clinical utility of BSI has recently been validated as a prognostic imaging biomarker. However, the clinical utility of the on-treatment change in BSI is dependent on the reproducibility of bone scans. The objective of this prospective study is to evaluate the intra-patient reproducibility of two bone scan procedures performed at a one-week interval.

**Methods:**

We prospectively studied prostate cancer patients who were referred for bone scintigraphy at our centres according to clinical routine. All patients underwent two whole-body bone scans: one for clinical routine purposes and a second one as a repeated scan after approximately one week. BSI values were obtained for each bone scintigraph using EXINI bone^BSI^ software.

**Results:**

A total of 20 patients were enrolled. There was no statistical difference between the BSI values of the first (median = 0.66, range 0–40.77) and second (median = 0.63, range 0–22.98) bone scans (*p* = 0.41). The median difference in BSI between the clinical routine and repeated scans was − 0.005 (range − 17.79 to 0). The 95% confidence interval for the median value was − 0.1 to 0. A separate analysis was performed for patients with BSI ≤ 10 (*n* = 17). Differences in BSI were smaller for patients with BSI ≤ 10 compared to the whole cohort (median − 0.1, range − 2.2-0, 95% confidence interval − 0.1 to 0).

**Conclusions:**

The automated BSI demonstrated high intra-individual reproducibility for BSI ≤ 10 in the two repeated bone scans of patients with prostate cancer. The study supports the use of BSI as a quantitative parameter to evaluate the change in total tumour burden in bone scans.

## Background

Bone metastases cause much of the morbidity and mortality associated with prostate cancer and lead to complications that include pathologic fractures and severe pain [1, 2]. Therefore, bone-targeted therapies are important and in continuous development. Over the last few years, several novel agents have been approved by the US Food and Drug Administration for use in advanced prostate cancer [3]. Despite the advances, several areas of urgent need remain. In the context of medical imaging more objective methods are needed for the evaluation of bone metastasis, staging, and response measures. Advances in this area would be valuable in clinical routine and clinical trials.

Bone scan examination remains the most widely used and recommended method for assessing metastatic spread to the bone when progression of the disease is suspected in patients with prostate cancer [4]. However, there is still no standardisation of bone scan interpretation. The current methods used in clinical routine are based on traditional visual analysis, which is qualitative and mainly focuses on merely whether or not metastatic lesions are present in the bone [4]. However, several studies have shown that the degree of tumour extension in the bone is a more accurate approach for prognostic evaluation. Nevertheless, in clinical practice, estimating the degree of tumour extension is also subjective and highly dependent on the interpreter [5]. Therefore, automatic quantitative analysis of the images could be useful for reducing intra- and inter-observer variability [6].

The Bone Scan Index (BSI) was developed and later automated to obtain more information from bone scans [7]. The index represents the percentage of bone affected by tumours and is calculated from bone scan images. BSI has been proposed as a pre- and post-treatment prognostic imaging biomarker as a complement to traditional clinical prognostic parameters to improve the stratification of patients [8, 9]. Several studies also discuss the possibility of using this biomarker as a predictive marker for treatment response since changes in BSI after follow-up have been related to survival and other outcomes [10, 11].

However, knowledge is needed about the degree of reproducibility of BSI measurements for the reliable detection of changes over time. Before BSI can be applied for therapy monitoring in clinical practice and clinical trials, the accuracy and precision of the measurement method and the spontaneous variability of the biological signal should be determined. BSI quantification shows robust reproducibility when analysing the same image [12], but the intra-patient variability when the patient is examined at different times is currently unknown (i.e. the variability of repeated measurements after re-injection of the compound within the same week) [13]. To our knowledge, test-retest data has not been published for the variability of BSI in patients with prostate cancer. Such an evaluation would clarify the biological reproducibility of BSI within each individual. Thus, the aim of this study is to assess the intra-individual reproducibility of automatically obtained BSI when measuring tumour burden in the bones of prostate cancer patients. The ultimate goal is to fully validate the automated BSI method as a clinical applicable biomarker.

## Methods

### Patients

The study participants were recruited from all prostate cancer patients who underwent bone scintigraphy at Sahlgrenska University Hospital in Gothenburg, Sweden, from March to October 2015 and at Skåne University Hospital, Sweden, from November 2015 to March 2016. The eligibility criteria for participating in the study included a documented prostate cancer diagnosis and age older than 70 years. The exclusion criteria included planned radiotherapy during the week after the first scan.

Those who met the inclusion criteria were asked to contribute an additional whole-body bone scan examination one week after the first bone scan examination. Due to the limited availability for performing additional examinations, patients were only asked to participate when a free camera time slot was available. The study was performed in accordance with the Declaration of Helsinki and was approved by the Regional Ethical Review Boards at Lund University, Sweden and the Regional Radiation Protection Committees at Skåne University Hospital and Sahlgrenska University Hospital. The patients gave written consent to participate.

### Bone scintigraphy

All patients were scanned according to the same standards used in clinical routine, as indicated in the current procedure guidelines for tumour imaging of the European Association of Nuclear Medicine [14]. Each participating patient underwent two whole-body bone scans: one performed as part of the clinical routine and a repeated bone scan performed approximately one week after. The bone scans were performed approximately three hours after intravenous injection of 600 MBq of technetium-99 m hydroxyethylene diphosphonate (Malmö) or technetium-99 m 2,3-dicarboxypropane-1,1-disphosphonate (Gothenburg). Anterior- and posterior-view whole-body images were obtained using one of four different gamma cameras: a Tandem Discovery 670 (GE Healthcare); Infinia (GE Healthcare); IRIX (Marconi Medical Systems), or Symbia (Siemens Healthcare).

The first three gamma cameras were used in Gothenburg, and the last one was used in Malmö. We aimed to use the same camera to examine the patients both times. All gamma cameras were equipped with low-energy, high-resolution, parallel-hole collimators with a scan rate of 10 cm/min and a 256 × 1024 matrix. Energy discrimination was provided by a 20% window for the first two cameras and 15% for the second two cameras. For all cameras, the energy discrimination was centred at 140 keV for Tc-99 m. All the resulting bone scan images showed the same quality level and were appropriate for further analysis.

### BSI analysis

BSI was calculated using the commercially available software EXINI bone^BSI^ version 2 (EXINI Diagnostics AB, Lund Sweden). The automated method of calculating BSI has been described in detail elsewhere [7] and was analytically validated in a recent study [12]. In summary, different anatomical regions of the skeleton were segmented, and hotspots were detected and classified as metastatic lesions or not. The mass fraction of the skeleton was calculated for each metastatic hotspot, and the BSI was finally calculated as the sum of all fractions. Only minimal manual corrections were made in cases of misclassification of the urine bladder or catheters as hotpots according to the manufacturer’s instructions.

### Statistical analysis

The intra-individual reproducibility of the repeated BSI measurements was tested using the Wilcoxon signed-rank test based on the bone scan measurements. The Bland-Altman method was used to detect systematic differences between the test-retest BSI measurements and to identify possible outliers. A linear regression procedure was performed to determine the presence of proportional bias.

Previous studies indicated high variability in comparisons of manual versus automatic BSI measurements in patients with extensive bone disease (BSI > 10) [7]. Therefore, we performed a second series of reproducibility analyses that included only patients who showed BSI < 10 in the clinical routine scan. Statistical significance was set at 0.05 for the tests performed. All statistical analyses were performed using IBM SPSS for Windows version 23.

## Results

A total of 20 patients were included (13 in Gothenburg and 7 in Malmö). The median age was 76 years (range 70 to 86 years). Table 1 provides the basic characteristics of the patients, the time between bone scans, and the time from injection to image acquisition.

**Table 1:** Basic characteristics of patients and scans

Age, year, median (range)	76 (70–86)
Time between bone scans, days, median (range)	6 (1–9)
1st Bone Scan: Time from injection to image acquisition, min (SD)	210 (25)
2nd Bone Scan: Time from injection to image acquisition, min (SD)	201 (30)

**Fig. 1:**
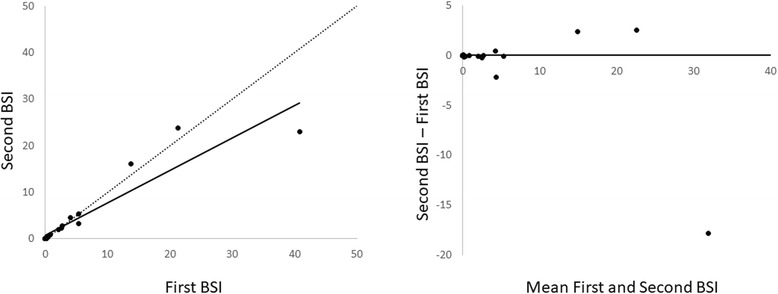
Scatter plot (left) and Bland Altman plot (right) for the whole cohort (*n* = 20). The regression line is solid, and the identity line is dotted in the left figure. In the right figure, the solid line represents the median

**Fig. 2:**
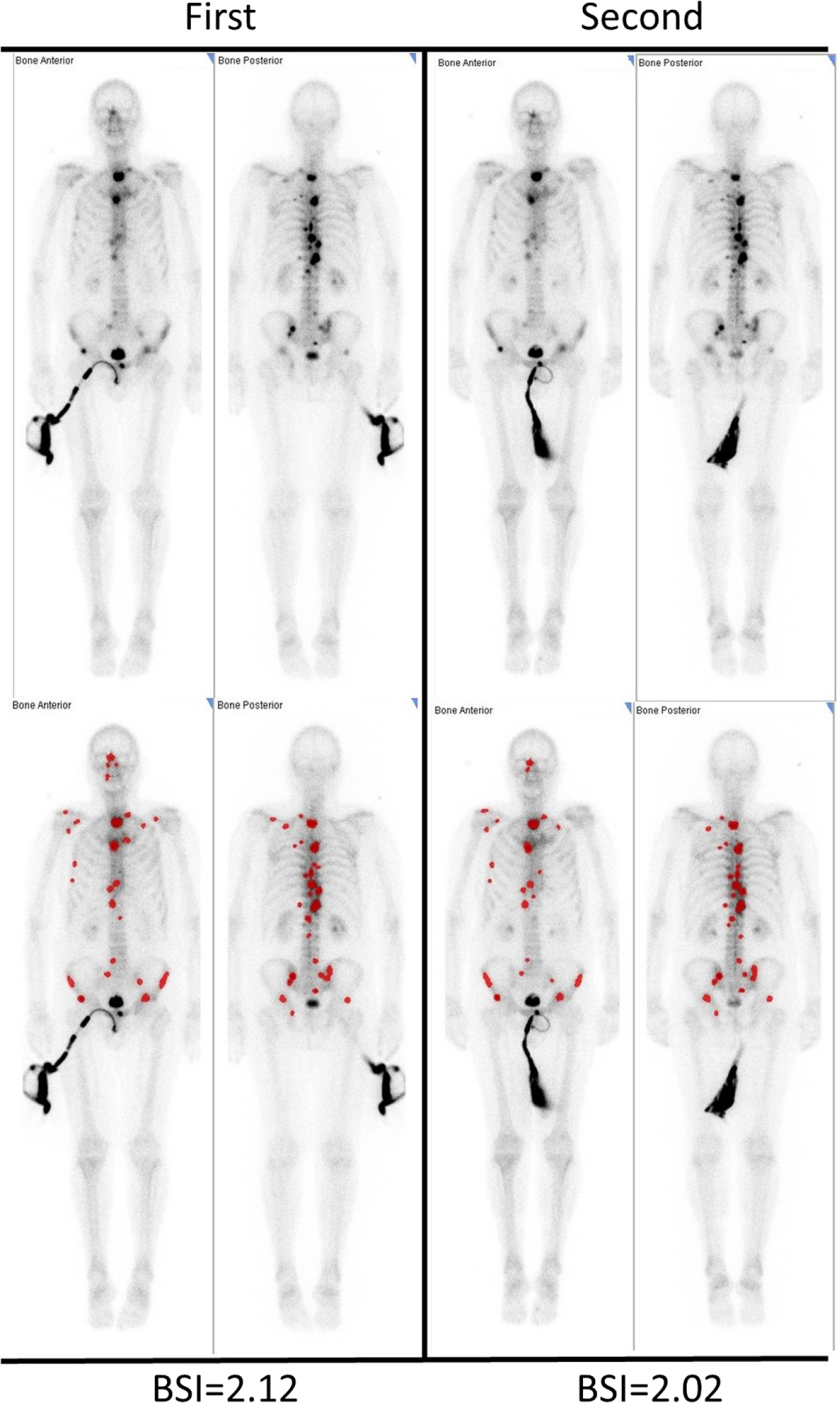
A patient with a small difference in BSI at two different examinations performed 6 days apart. The upper row shows the bone scan (anterior and posterior views) after the first (left) and second (right) examinations. The lower row shows the hotspots automatically detected by the EXINI software. Blue lesions are not considered metastatic (and thus not included in the BSI measurement), and red lesions are considered metastatic

**Fig. 3:**
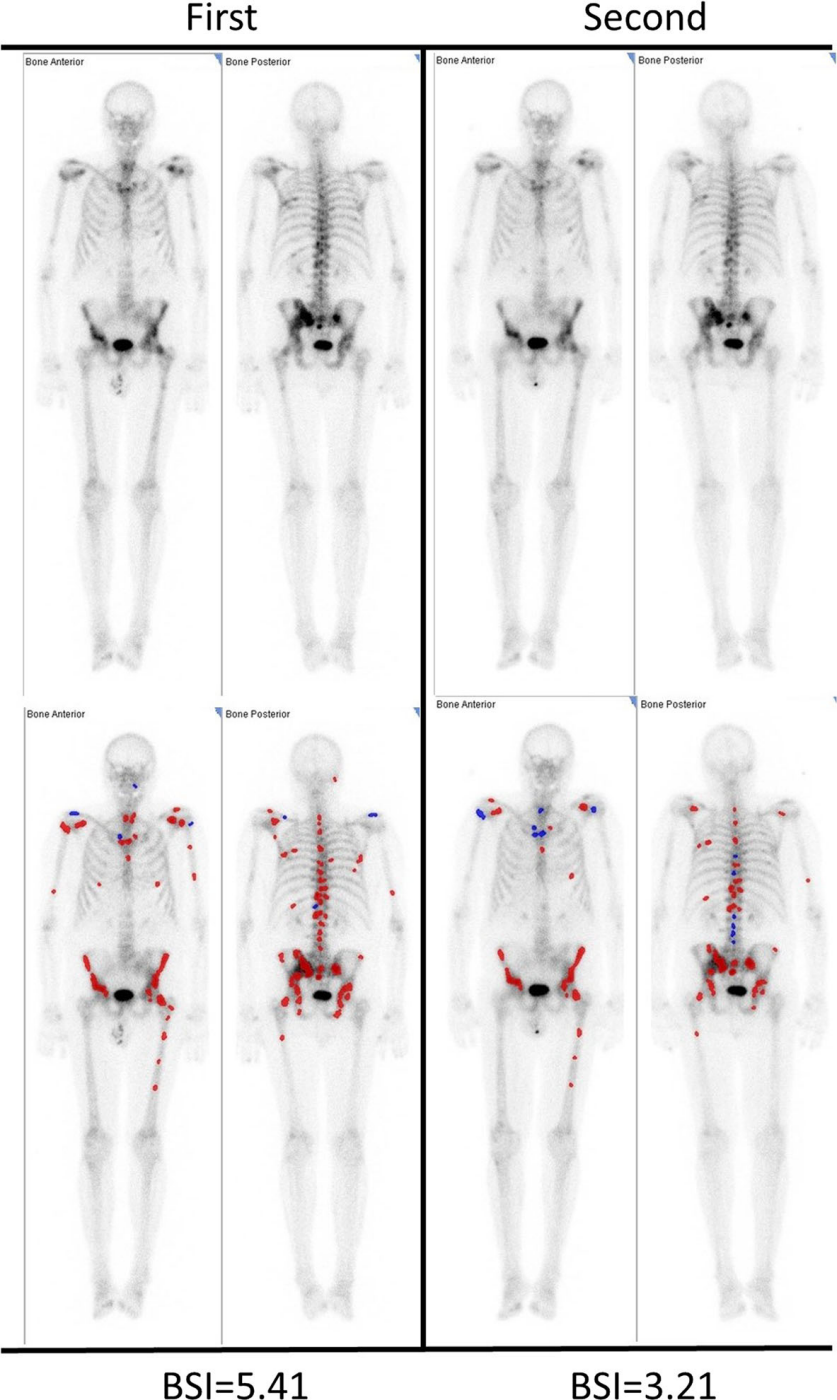
A patient with a large difference in BSI at two different examinations performed 6 days apart. The upper row shows the bone scan (anterior and posterior views) after the first (left) and second (right) examination. The lower row shows the hotspots automatically detected by the EXINI software. Blue lesions are not considered metastatic (and thus not included in the BSI measurement), and the red lesions are considered metastatic

**Fig. 4:**
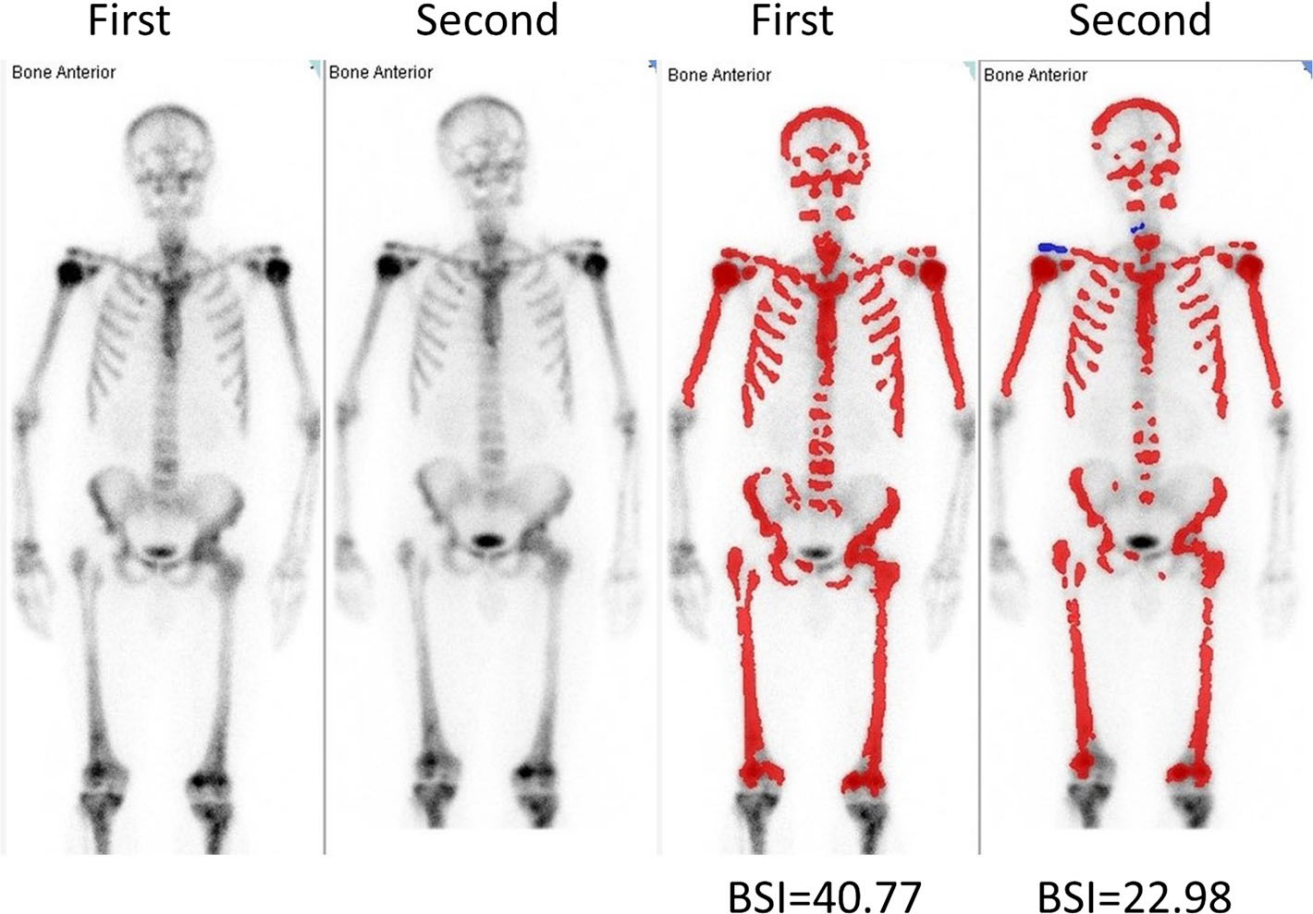
The patient with the largest difference in BSI at the two different bone scans. The figure shows anterior views after the first and second examination (left images). The right images show the hotspots automatically detected by the EXINI software. Blue lesions are not considered metastatic (and thus not included in the BSI measurement), and the red lesions are considered metastatic

The BSI values for the whole cohort had a median value of 0.66 (range 0 to 40.77) for the first BSI measurement and 0.63 (range 0 to 22.98) for the second BSI measurement. There was no significant difference between the routine and repeated BSI measurements (*p* = 0.41). The median difference in BSI for the whole cohort (*n* = 20) was − 0.005 (range − 17.99 to 0) with a 95% confidence interval (CI) of − 0.1 to 0 (Table 2). The scatter and Bland-Altman plots are presented in Fig. 1. For five of the patients, BSI was identical in both examinations. Eight patients showed an absolute difference in BSI of > 0 to 0.1, two patients had an absolute difference of > 0.1 to 0.5, and four patients had an absolute difference of > 1. Figure 2 shows a patient with similar BSI at the two examinations, and Fig. 3 shows a patient with a large difference in BSI between the two different examinations. The largest difference in BSI between the first and second bone scan was found in the patient with highest BSI (Fig. 4).

**Table 2:** Median and range for BSI measurements from the first and the second bone scans

All patients (*n* = 20)			
First BSI	Second BSI	BSI difference	p
0.66 (0–40.77)	0.63 (0–22.98)	−0.005 (−17.99–0)	0.41 (NS)
Patients with BSI ≤10 (n = 17)			
First BSI	Second BSI	BSI difference	p
0.24 (0–5.41)	0.14 (0–5.28)	−0.1 (−2.2–0)	0.11 (NS)

*BSI* Bone Scan Index, *NS* Not significant

**Fig. 5:**
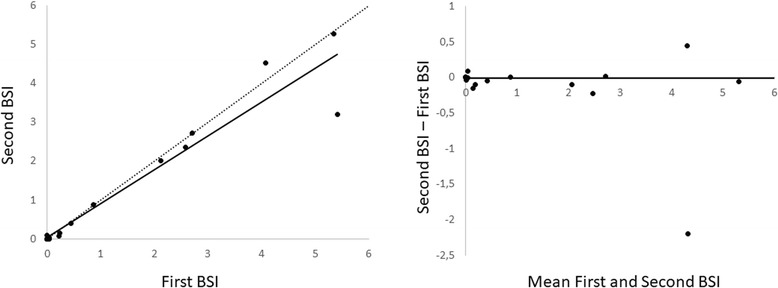
Scatter plot (left) and Bland Altman plot (right) for patients with BSI < 10 (*n* = 17). The regression line is solid, and the identity line is dotted in the left figure. In the right figure, the solid line represents the median

The calculations were also performed for patients with BSI ≤ 10 at the first examination (Table 2). A total of 3 of the 20 patients were therefore excluded from the second series of analyses, leading to a sub-cohort of 17 patients. All three excluded patients had a difference > 1 between the first and second BSI. The BSI values for this sub-cohort had median values of 0.24 (range 0 to 5.41) for the first BSI measurement and 0.14 (range 0 to 5.28) for the second BSI measurement. There was no significant difference between the routine and repeated BSI measurements (*p =* 0.11). The median difference in BSI for the sub-cohort was − 0.01 (range − 2.2 to 0) with a 95% CI of − 0.1 to 0. The scatter and Bland-Altman plots are presented in Fig. 5.

## Discussion

This study shows high intra-individual reproducibility in the BSI values calculated from bone scans with only minimal manual intervention for different bone scans taken one week apart, especially for patients with BSI ≤ 10. The median difference in BSI for the whole cohort was − 0.005 with a 95% CI of − 0.1 to 0. These results show that the automated BSI is a consistent measure of tumour burden in the bones of prostate cancer patients. For patients with a high BSI the method is less reproducible. As seen in Fig. 4, small differences in hotspot delineation in a patient with a highly metastasised skeleton lead to a vast difference in BSI.

The clinical implications of these results are related to the utility of using BSI difference measurements when analysing follow-up bone scans to describe significant changes in bone status in a quantitative and more objective manner. This information could be useful as a complement to traditional visual analysis of bone scans to produce more objective reports regarding possible stabilisation or progression of bone disease. This type of evaluation would be valuable when monitoring patients undergoing specific therapies for prostate cancer to support physicians on decisions to adjust treatment when necessary.

A recent meta-analysis examined the prognostic value of BSI as an imaging biomarker in prostate cancer [15]. The analysis included 14 high-quality studies involving 1295 patients. The pooled results indicated that a high baseline BSI and high change in BSI over time were significantly predictive of poor overall survival and that BSI could improve predictive models. The conclusion was that BSI may be beneficial as a predictive imaging biomarker in patients with metastatic prostate cancer.

Previous studies have explored the reproducibility of BSI when analysing bone scans performed at different times after injection [16]. Other studies showed robust reproducibility when analysing the same image [12], but the intra-patient variability is currently unknown (i.e. the variability of a repeated measurement after re-injection of the compound within the same week). Therefore, the design of the present study included a completely new bone scan examination conducted approximately one week after to explore the intra-individual reproducibility of automated BSI.

Among the limitations of this study are those of the bone scan technique itself and those related to daily clinical work. One example is the impossibility of performing the repeated bone scans using exactly the same gamma camera in some cases, which occurred for three patients. However, Anand et al. [17] did not find any significant difference in BSI in simulated phantom data for gamma cameras from different vendors. Considering the high variability between manual and automated BSI measurements in patients with BSI values > 10 [7], we included a second series of analyses for such patients. The difference between the first and second BSI was higher for patients with BSI > 10.

Despite the small scale of this prospective study, we have presented important evidence in support of the hypothesis of high intra-individual reproducibility of the automated BSI measurement method. Reproducibility studies are rare in the field of nuclear medicine, partly due to the need for an extra dose of radiation to obtain subsequent imaging studies. In this study, the risks associated with extra radiation are minimal, considering the patients’ age. This examination was needed for further standardisation for evaluating changes in BSI change [13, 18, 19]. The results also indicate the possibility of using this method as a clinically applicable biomarker in patients with prostate cancer.

## Conclusions

Automated BSI demonstrated high intra-individual reproducibility for BSI ≤ 10 in the two repeated bone scans of prostate cancer patients. The study supports the use of BSI as a quantitative assessment to evaluate changes in total tumour burden in bone scans.

## Abbreviations

BSI: Bone Scan Index
CI: Confidence interval
